# Review of Endometrial Receptivity Array: A Personalized Approach to Embryo Transfer and Its Clinical Applications

**DOI:** 10.3390/jpm13050749

**Published:** 2023-04-27

**Authors:** Sarah C. Rubin, Mawerdi Abdulkadir, Joshua Lewis, Aleksandr Harutyunyan, Rahim Hirani, Cara L. Grimes

**Affiliations:** 1 School of Medicine, New York Medical College, 40 Sunshine Cottage Road, Valhalla, NY 10595, USA; 2 Department of Obstetrics and Gynecology and Urology, New York Medical College, Valhalla, NY 10595, USA

**Keywords:** endometrial receptivity array, in vitro fertilization, pre-implantation testing, frozen embryo transfer, hormone replacement therapy, implantation failure

## Abstract

Successful outcomes of in vitro fertilization (IVF) rely on both the formation of a chromosomally normal embryo and its implantation in a receptive endometrium. Pre-implantation genetic testing for aneuploidy (PGT-A) has been widely accepted as a tool to assess the viability of an embryo. In 2011, the endometrial receptivity array (ERA) was first published as a tool to determine when the endometrium is most receptive to an embryo, commonly referred to as the “window of implantation” (WOI). The ERA uses molecular arrays to assess proliferation and differentiation in the endometrium and screens for inflammatory markers. Unlike PGT-A, there has been dissent within the field concerning the efficacy of the ERA. Many studies that contest the success of the ERA found that it did not improve pregnancy outcomes in patients with an already-good prognosis. Alternatively, studies that utilized the ERA in patients with repeated implantation failure (RIF) and transfer of known euploid embryos demonstrated improved outcomes. This review aims to describe the ERA as a novel technique, review the various settings that the ERA may be used in, such as natural frozen embryo transfer (nFET) and hormone replacement therapy frozen embryo transfer (HRT-FET), and provide a summary of the recent clinical data for embryo transfers in patients with RIF utilizing the ERA.

## 1. Introduction

In vitro fertilization relies on the success of a multi-fold process which includes both the creation of a normal chromosomal embryo and the implantation of the embryo into a receptive endometrium [1,2]. Owing to the development of precision medicine and technology in the field of reproductive endocrinology, there are tools that can be used to aid in both of these processes. As for the creation of a chromosomally normal embryo, pre-implantation genetic testing for aneuploidy (PGT-A) has been accepted within practice guidelines as the definitive tool to be used to test for whether an embryo is euploid or aneuploid [3]. While PGT-A can be used in all patients, it has been shown to offer a benefit particularly for patients experiencing recurrent implantation failure (RIF) and severe male infertility [4,5]. For the implantation of an embryo into a receptive endometrium, the ERA is a tool that was developed to determine when the endometrium is most receptive to an embryo [6].

Endometrial receptivity has been defined in the literature as the period of endometrial maturation during which the trophectoderm of the blastocyst can attach to the endometrial epithelial cells and subsequently invade the endometrial stroma and vasculature [7]. Endometrial maturation is in part accomplished by exposure to steroid hormones such as estrogen in the follicular phase, and progesterone in the luteal phase [8]. In the follicular phase, estrogen signals induce the proliferation of the endometrial lining, consequently causing an increase in progesterone receptor expression [9]. Following ovulation, progesterone further changes the endometrium to create an environment receptive to implantation and ultimately maintain an early pregnancy [8].

In addition to hormones, many molecular pathways including adhesion molecules, cytokines, and growth factors work synchronously to create a window of implantation (WOI) [10]. This WOI is a short period of optimal endometrial receptivity and typically occurs between days 20 and 24 of a normal 28-day menstrual cycle.

However, endometrial receptivity exists on a spectrum and is not necessarily “all-or-nothing” [10]. When the WOI is not achieved or receptivity is non-optimal, mild defects may occur, including placental abnormalities which lead to preeclampsia or a low birth weight. However, more severe forms can lead to infertility and recurrent pregnancy loss.

In an effort to decrease these aforementioned poor outcomes, the ERA was developed as a tool to pinpoint the WOI, thus maximizing endometrial receptivity and improving successful embryo implantation rates [6]. In the wake of this tool, there is dissent toward its utility in improving outcomes for infertile patients. This review aims to describe the ERA as a novel technique and review the various settings in which the ERA may be used, including natural frozen embryo transfer (nFET) and hormone replacement therapy frozen embryo transfer (HRT-FET). Further, we aim to provide a summary of the recent clinical data for embryo transfers in all patients using the ERA, in addition to data of specific populations including those with pregnancy problems such as RIF, adenomyosis, and endometriosis. To the best of our knowledge, this review is the first of its kind to synthesize the clinical data available on the ERA in conjunction with the literature available on the technology itself. The key point we aim to highlight in this review article is that the endometrial receptivity factor in the treatment of infertility is often neglected. In doing so, the personalized approaches to improving success from the endometrial perspective are neglected as well. Thus, we aim to shed light on a novel technique, and the settings in which there has been demonstrated success in its use.

## 2. A Description of Endometrial Receptivity Array

While the ERA is a novel technique, the concept of endometrial receptivity is not an entirely new idea. In 1975, Noyes et al. identified morphological changes in the endometrium throughout the menstrual cycle. This laid the groundwork for the development of further tools investigating endometrial receptivity [11]. There are currently three tests for endometrial receptivity that are commercially available: 1. the endometrial function test (EFT) which consists of both a histological assessment and endometrial development, 2. the BCL6 test which evaluates inflammatory markers for endometriosis, and 3. the ERA which “dates” the endometrium [6]. While the EFT has promising clinical results, due to its time- and labor-intensive nature and the expertise necessary for assessment using it, this tool is recommended for specific cases in which further embryo creation is not possible, or in which a small number of embryos are being transferred [6,12,13]. The BCL6 test has similarly shown promising outcomes as well; however, it is specific to endometriosis, and therefore is not generalizable to all patients experiencing infertility [14,15,16]. Due to the limited context in which the EFT and BCL6 test can be applied, the remainder of this review will be dedicated to examining the ERA in detail, as our primary focus of investigation. This emphasis solely on the ERA is necessary as there is dissent in the field toward the efficacy of the ERA and when it is used—unlike the views on other endometrial receptivity tests previously mentioned. Thus, by highlighting the function of the ERA and its application in various patient populations, we aim to elucidate when this tool may be best applied.

The endometrial receptivity array (ERA) was first developed in 2011 by Díaz-Gimeno et al. in an effort to define the transcriptomic signature of human endometrial receptivity [6,17]. The research fueling the development of this tool was in large part enabled by the completion of the Human Genome Project in 2001 [18,19]. The Human Genome Project laid the groundwork for microarray-based gene expression technology and transcriptomic analysis. This technology, when applied to the endometrium, has allowed further research investigating transcriptomic expression changes throughout the menstrual cycle and within the context of different gynecologic conditions [20,21]. Many studies have shown that during the menstrual cycle, and more specifically the WOI, the endometrium has a specific transcriptomic profile [21]. This transcriptomic profile is thought to be consistent with a “receptive phenotype”, meaning that the differential expression of genes during the WOI may create an endometrial environment more conducive to successful implantation [19].

In the development of the ERA, Díaz-Gimeno et al. selected genes involved in human endometrial receptivity as the basis of this tool’s analysis. To do so, they analyzed the entire human genome, highlighting the different gene expression profiles in a receptive endometrium and a pre-receptive endometrium. This was carried out by utilizing expression data for these genes collected during the group’s previous work. The genes that were shown to have an absolute >3-fold change and a false discovery rate of <0.05 in a receptive endometrium were selected. In total, 238 genes were identified as differentially expressed at the receptive phase and pre-receptive phase. Using these selected genes, they built an Agilent customized gene expression microarray (Madrid, Spain) known as the ERA. To characterize the nature of these genes, they performed a functional analysis to determine if the selected genes were over-represented in biological functions, including processes relating to the immune system, circulation, or the response to external stimulus. Some of the over-represented terms included oxidoreductase activity, receptor binding, and carbohydrate binding. Consequently, clustering sample analysis was performed, grouping samples as either “receptive” or “nonreceptive.” This clustering was carried out to detect genes with similar behavior to those in other phases of the menstrual cycle. For the endometrial transcriptomic signature, a comparison between the differential expression of the genes that were receptive and pre-receptive were compared and the genes that were receptive and proliferative were compared. The intersection of these comparisons generated a list of 134 statistically differentially expressed genes in the receptive group compared to the expression of both proliferative and pre-receptive genes [17]. To prove the translational efficiency of the ERA, the group then designed a bioinformatic test with predictive power to define the endometrium gene expression profile compatible with LH +7. The predictor showed a specificity of 0.8857 and sensitivity of 0.99758 for endometrial dating, thus demonstrating the efficacy of this tool at objectively diagnosing the endometrial receptivity status. For pathological classification, there was a specificity of 0.1571 and a sensitivity of 0.995, attributed by the authors to the difficulty in obtaining samples; nonetheless, it should be noted that none of the normal samples were labeled as pathological. While the ERA may not be a tool used primarily for pathological classification, the data makes evident that the ERA demonstrates specificity and sensitivity as a tool for endometrial receptivity.

While there are many complex mechanisms at play in defining the transcriptomic signature of endometrial receptivity, all that is required of the provider and patient from a clinical standpoint is an endometrial biopsy. The nuances of the timing of the biopsy will be described in later sections. Once the biopsy is obtained, it is sent to the laboratory for processing; the ERA begins by dissolving the biopsy specimen for molecular analysis using the differential expression of 238 genes, as described above. In dissolving the biopsy, there is no histological assessment of the patient’s endometrium; however, it is possible to perform a concurrent endometrial biopsy for a pathological review separate from the ERA. Within the scope of the ERA performed alone, RNA is extracted from the biopsy, and processed using the microarray, as described previously. The array is coupled to a computational predictor that is able to identify the receptivity status of an endometrial sample and diagnose the personalized WOI (pWOI) of a given patient.

In 2013, Díaz-Gimeno et al. further compared the accuracy and reproducibility of the ERA to standard histological methods. They found that the ERA is more accurate than histological dating, is completely reproducible, and can determine the pWOI regardless of the sample’s histologic appearance [17,22]. While studies show that WOI length, approximately two days, is largely constant amongst women, it is displaced in 20% of the population and in ¼ of women with RIF [23]. Thus, the ERA allows the identification of a personalized WOI which can then be applied to a patient’s FET plan, so that the medication and transfer timing are determined by when the endometrium is most receptive [17]. An ERA is flexible in this sense as it may be used in both hormone replacement therapy frozen embryo transfer (HRT-FET) and natural FET (nFET). In each case, minor adjustments must be made for it to be compatible with each cycle [23]. The specifics distinguishing these two cycles will be discussed further in later sections. Through the development of the ERA, it is evident that endometrial receptivity can be identified on a transcriptomic level. It is with this analysis that we can better identify each patient’s personalized WOI and harness this knowledge to optimize IVF outcomes.

## 3. ERA in the Context of Natural Frozen Embryo Transfer

Prior to discussing the application of the ERA in FETs, it is important to consider the development of FET and the different FET protocols available. With improvements in embryo cryopreservation technology and the rise in single embryo transfers, the rate of FETs has risen dramatically compared to that of fresh embryo transfer. The increase in single embryo transfers means that there is a higher number of quality embryos available for cryopreservation and potential future use [24]. This is in part an effort- and cost-saving measure in the case of a fresh embryo transfer failing, allowing a backup supply of frozen embryos to be available. These improvements have allowed FET success rates to rival those of fresh embryo transfer, establishing FET as an essential component of IVF treatment [25].

Paralleling the rise in FET is a growing body of evidence suggesting that pregnancy rates, as well as neonatal and perinatal outcomes, are improved when using FET rather than fresh embryo transfer [26,27]. FET has been shown to have lower rates of preterm birth and low birth weight compared to fresh cycles [28]. Additionally, there is no evidence of an increase in stillbirths or congenital malformations when using FET [25,28]. The worse outcomes associated with fresh embryo transfer are thought to be due in part to supraphysiologic estrogen and progesterone levels following the use of hCG to stimulate ovulation [29]. The rapid elevation of E2 and progesterone causes a rapid transition from a proliferative to secretory endometrium, thus “expediting” the endometrial preparedness following egg retrieval. In a fresh cycle, where embryo transfer is performed in the same cycle as egg retrieval is, this expedited progression of the endometrial lining will cause the WOI to occur earlier in the cycle; thus, the development of the embryo and endometrial receptivity will be out of sync.

Endometrial receptivity is not an all-or-none phenomenon, but implantation outside of the optimal window may lead to an increased incidence of placental abnormalities, influencing the outcomes of the pregnancy [30]. By using FET, the embryos are frozen for use in subsequent cycles, thus avoiding supraphysiologic hormone levels and an asynchronous implantation window, leading to better outcomes than those seen in fresh transfers. Furthermore, using FET whether it be HRT-FET or nFET allows us to maintain a higher degree of control over the uterine lining as we are able to plan and prepare from the beginning of the cycle [31]. While this may include the administration of medications in some cases, the fine-tuning this provides in preparing the endometrium makes the use of the ERA possible. An ERA is only able to be used in the context of a FET, as will be further explicated in the following sections.

HRT-FET and nFET are the two main available protocols for FET. nFET relies on the body’s endogenous hormones to drive the menstrual cycle, followed by oocyte collection, fertilization, and FET. nFET may be considered for women who have regular menstrual cycles as it involves fewer medications and therefore has a lower cost [32]. nFET can be further divided into two subtypes: modified-nFET and true-nFET.

True nFET typically begins with transvaginal ultrasonography (TVUS) on days 2–3 of menses. This is carried out to rule out the presence of any cysts or a persistent corpus luteum from a previous cycle, either of which may interfere with the current cycle [33]. Between cycle days 7–10, TVUS monitoring continues to measure the size of the leading follicle. Blood work is performed to monitor when the LH surge begins and for ovulation shortly after [34]. Embryo transfer is scheduled for 6 days post-LH surge (LH +6). After the embryo has been transferred, luteal phase support (LPS) in the form of exogenous progesterone may be given for 8–10 weeks to support the endometrium. This is carried out to provide progesterone in place of the corpus luteum until the placenta can begin producing adequate amounts on its own, but is not always necessary [35].

Modified nFET follows the same general protocol, however, once the leading follicle has reached a diameter of 16–20 mm, hCG is given to induce ovulation. In a modified nFET cycle, embryo transfer is typically scheduled 7 days post-hCG administration (hCG +7) [33]. The benefit of the modified nFET cycle is that by artificially inducing ovulation, there is less difficulty in detecting it than there is in true nFET. This is beneficial to both the patient and provider, as it often requires less monitoring, has a decreased likelihood of missing ovulation causing a need to cancel the cycle, and allows a certain degree of planning the timing of embryo transfer. Additionally, the exogenous hCG will induce progesterone production to support the endometrium acting as its own form of LPS. The hCG trigger carries the risk of causing an inappropriately elevated progesterone level and as a result, an asynchronous WOI. To minimize this, the lowest necessary dose of hCG should be given [33].

**Figure 1:**
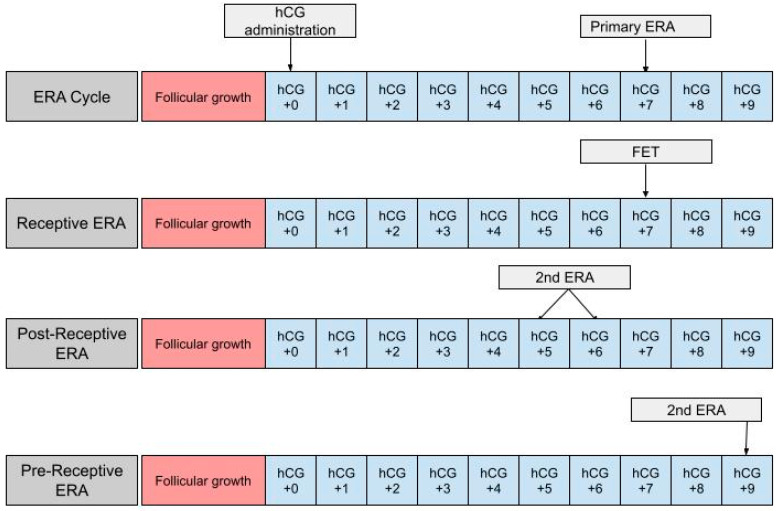
The first row shows the timing of the ERA biopsy in a modified natural cycle. hCG +0 is the day of the hCG trigger shot. The second row shows the timing of the frozen embryo transfer (FET) if the ERA yields a receptive result. The final two rows show the timing of the second ERA biopsy if the result of the initial ERA is post-receptive or pre-receptive, respectively.

The ERA can be used in a modified nFET cycle in an attempt to better guide the timing of embryo transfer to best match the WOI. The ERA cannot be used with true nFET as even slight cycle–cycle variations in the detection or time of ovulation can be enough to render an ERA report inaccurate. Administration of hCG is necessary in order to time embryo transfer to when the endometrium is most receptive. In an ERA, an endometrial biopsy is performed in a separate cycle, prior to embryo transfer [36] (Figure 1). The biopsy is taken at either LH +7 or hCG +7, which are considered to be equal under the ERA protocol, and sent to Igenomix (Miami, FL), the medical laboratory that produces, distributes, and analyzes the ERA [37]. A manual describing how to analyze the ERA report is included on the Igenomix website for provider use. The details highlighted in the manual are described as follows: the results of the ERA will come back as receptive or nonreceptive. If receptive, then the time in the cycle that the biopsy was performed represents the optimal WOI and embryo transfer should be performed at the same time (±3 h) in the subsequent cycle. Nonreceptive results are given as pre-receptive or post-receptive and may give a predicted WOI [38]. If the result is post-receptive, or if it is prereceptive by 2 or more days, a second biopsy and ERA may be needed in order to validate the predicted WOI [39]. The biopsy will need to be performed at LH +5 or LH +6 if the result is post-receptive or LH +9 if the result is pre-receptive [23]. The ERA has been shown to be highly consistent between menstrual cycles, so the optimal WOI measured in the previous cycles should remain stable in subsequent cycles. This consistency makes the ERA more reliable than histologic analysis [22].

## 4. ERA in the Context of Hormone Replacement Therapy Frozen Embryo Transfer

Hormone replacement therapy (HRT) is the other main protocol used in frozen embryo transfer (FET). In a HRT-FET cycle, exogenous estradiol (E_2_) and progesterone (P) are administered at set points to enhance one’s natural cycle, optimizing the endometrium for transfer [33]. The goal of HRT is to prepare the endometrium for FET, but there is some variation in the protocols used. The HRT cycle begins with the administration of E_2_ on day one, two or three. E_2_ participates in the proliferation of the endometrium and the suppression of spontaneous follicle growth. E_2_ can be administered as a fixed 6 mg daily dose or in increasing increments that start at 2 mg/day and increase up to 6 mg/day [40]. There is also variability in the routes of E_2_ administration, including oral, vaginal, and transdermal routes. The variations in dose and route of administration have been shown to produce comparable outcomes [41]. Starting on day 12, a transvaginal ultrasound (TVUS) is used to assess whether the endometrium has reached the desired thickness of >7 mm and to confirm the absence of a leading follicle [42,43,44]. In the event of the presence of a leading follicle, the cycle is canceled, and FET cannot be performed until the following cycle. E_2_ administration can last up to 36 days, following which progesterone supplementation is commenced [45,46].

In contrast to E_2_, there is some debate in the literature regarding the dosing and routes of administration of progesterone [47]. It can be administered through oral, intramuscular, or subcutaneous routes, or as a suppository. However, a vaginal suppository remains the most widely used option for the administration of progesterone. Progesterone initiates the secretory transformation of the endometrium, preparing it for implantation [48,49]. It should be continued until a luteo–placental shift occurs and is generally continued until the 10th to 12th week of gestation [50]. Typically, the endometrium first has the possibility of being receptive on the third day after the initiation of progesterone [51]. It is the duration of progesterone that can be modified and ultimately optimized when an ERA is incorporated into a HRT-FET cycle.

**Figure 2:**
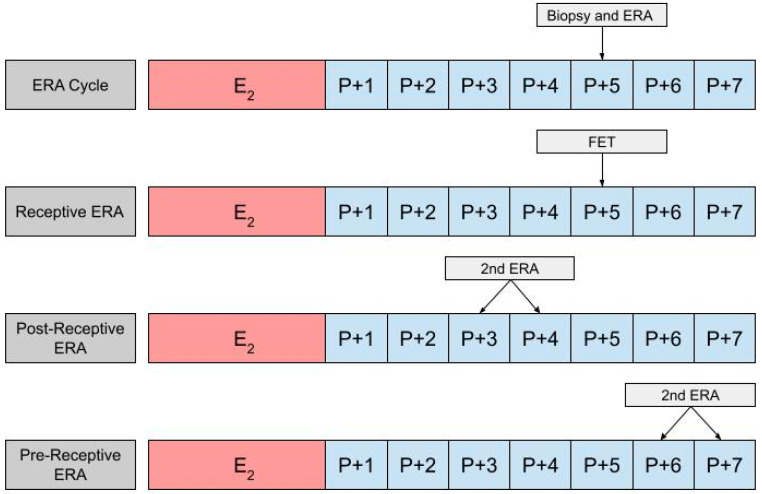
The first row shows the timing of the biopsy and the ERA in a cycle. The subsequent row shows the timing of the frozen embryo transfer (FET) if the ERA indicates a receptive endometrium. The final two rows show the timing of the second ERA if the result of the initial ERA is post-receptive or pre-receptive, respectively.

In using an ERA to guide the timing for an HRT-FET cycle, a patient will ultimately need two cycles, the first being an ERA cycle, and the second being an HRT-FET cycle [23,52,53]. In the ERA cycle, the patient uses the same medications they would in an HRT-FET cycle, mimicking the uterine conditions in an eventual transfer cycle (Figure 2). However, instead of embryo transfer occurring, an endometrial biopsy can be performed on day P +5 and sent for an ERA. The results of the ERA are used to personalize the date of the embryo transfer (and/or the initiation of progesterone) to the individual receptivity of each patient’s endometrium. Following the endometrial biopsy, the ERA report can return one of three results: a receptive, pre-receptive, or post-receptive result. If the ERA report concludes that the patient is receptive, then the endometrium is ready for embryo transfer and FET is performed on the day that the ERA is indicated as receptive, in the following cycle. If the result is nonreceptive, then gene profile analysis results reveal whether the endometrium is pre-receptive or post-receptive. In the next cycle, a second ERA is performed on day P +6 or P +7 if the report states that the patient was pre-receptive. Alternatively, the ERA is performed on P +3 or P +4 if the patient is considered post-receptive. If the patient is considered receptive following the second ERA, then FET is performed on the same cycle day that the ERA is reported to be receptive, in the following cycle [23]. While the above-mentioned process is the most accurate method with which to obtain the patient’s WOI, in clinical practice it is up to the discretion of the provider and patient whether or not to perform a second ERA at all, following the initial nonreceptive result. In some cases, embryo transfer us performed without confirming the receptiveness of the endometrium with a second ERA cycle.

Igenomix (Miami, FL) is the medical testing laboratory that creates and distributes the endometrial receptivity array. The provider sends the endometrial biopsy collected from the patient to Igenomix. Igenomix analyzes the endometrial biopsy using the technique described in an earlier section and reports the receptivity status of the endometrium. As described in the prior section, providers can access the guideline for the ERA through the Igenomix website, Igenomix.com. The guideline outlines the rationale, indications, and methodology behind the ERA. In this guideline, providers can find an example of an ERA result report along with a guide on how to interpret the results, which we have also summarized in the previous paragraph. Additionally, the company has created a calculator for clinicians to effectively use the results generated in the ERA report; it aids in determining the timing of progesterone administration and embryo transfer to optimize the patient’s receptivity. The “personalized Embryo Transfer (pET) Calculator,” available exclusively on the Igenomix website, allows clinicians to select the cycle type (HRT vs. nFET) and the type of the embryo (blastocyst vs. day 3 embryo) [54]. Following these selections, the provider can input the number of hours provided by the ERA report. Finally, the provider may select whether they would like to calculate the initiation time of progesterone based on a preferred FET time or calculate the time that FET should take place based on when progesterone therapy was started. For example, if a patient is undergoing HRT-FET and is transferring a blastocyst, the ERA may recommend a transfer time of 154 h after the initiation of progesterone. Using these parameters in the calculator and selecting a hypothetical FET date of 20 April 2023 at 9:00 a.m., the software calculates the exact date and time of the first progesterone intake as 13 April, 11:00 p.m. ± 3 h. Additionally, this tool can be used in modified nFET cycles but not in true nFET cycles. In modified nFET cycles, the ERA-recommended transfer time can be used to calculate a window for embryo transfer post-hCG administration or a hCG administration time based on a hypothetical future transfer date. The calculator can be extremely helpful for clinicians and should be recommended for the use of ERA with personalized FET.

## 5. Clinical Outcomes Using ERA in Infertile Women

The success of in vitro fertilization (IVF) has increased significantly from a clinical pregnancy rate of 6% to a live birth rate between 47% to 57% with frozen embryo transfer (FET) [53,55]. This is largely due to updated protocols and laboratory techniques including genetic screening of embryos for aneuploidy. Even with this advancement, implantation failures occur in previously screened embryos [56]. Hence, this has sparked interest in endometrial receptivity as a possible alternative to improve clinical pregnancy and live birth rates after FET. Thus, the literature assessing the success rate of ERA use prior to FET through clinical pregnancy and live birth rates were reviewed. This section describes studies that evaluate these metrics in largely unselected populations. In the following section, this will be contrasted with studies assessing clinical outcomes in the setting of specific patient populations including RIF patients, patients with adenomyosis, and patients with endometriosis (Table 1).

Hombalegowda and Zeigler [61] examined the benefit of ERA testing in women who had undergone their first FET from January 2017 to January 2019 compared to the outcomes of those who did not undergo ERA testing before implantation. In the control group, 224 patients underwent FET without ERA testing, while the other 72 patients in the study group underwent ERA testing before embryo transfer. Within the ERA testing group, 31 endometrial samples were receptive, 32 samples were pre-receptive, and 9 samples were post-receptive. The samples that were nonreceptive (pre-receptive or post-receptive) underwent further adjustments in order to determine the appropriate window of implantation for FET. After these changes were made, there was a small increase in the live birth rates with a 50% rate for the ERA group compared to a 39.7% rate in the control group (*p* = −0.13). Furthermore, there were lower miscarriage rates in the ERA group with a rate of 5.6% compared to a rate of 10.7% in the control group (*p* = −0.25). Despite these outcomes, the differences were not statistically significant. Hence, the study concluded that although there is a slight benefit of ERA use, including an increase in live birth rates and a decrease in miscarriage rates in patients with first-time FETs, the data were not significant. However, a notable limitation of this study is the absence of pre-genetic screening since aneuploidy could give rise to implantation failure [62,63]. Furthermore, the literature for the ERA without PGT-A of embryos is also limited. Thus, further studies should be conducted to determine whether or not genetic screening of embryos, that also undergo ERA testing prior to implantation, cause an increase in live birth rates.

Since patients with prior implantation failures have increased rates of aneuploid embryos, genetic screening for aneuploidy is essential [57]. Hence, we review studies that include PGT-A of embryos prior to FET. The following retrospective cohort study occurring between April 2016 and March 2017 examined the successful implantation rates of patients in their first through third embryo transfer, whose embryo transfer days were adjusted according to the results of the ERA, compared to the implantation rates of patients who did not undergo ERA testing [59]. The control group consisted of 503 patients who underwent FET cycles without prior ERA testing. Although 53 patients had undergone ERA testing, 12 of these patients did not go through FET; thus, only 41 patients underwent FET after ERA testing. To eliminate any confounding variables, both groups of patients were standardized for the following: age, previous embryo transfer number (first to third), endometrial thickness, transferred embryo number, and ongoing pregnancy rates. In order to increase the success of implantation in this study, patients were provided with preimplantation genetic screening. Specifically, more patients in the ERA testing group had genetic screening, with 34.1% of patients undergoing the screening compared to the control group, 14.7% of which underwent screening, with a *p*-value of 0.003. Despite these changes, however, the ongoing pregnancy rates were comparable, being 35.2% in the control group vs. 39% in the ERA group. With the ERA group specifically, out of the 53 patients who had ERA testing performed, 5 samples were post-receptive, 29 were prereceptive, and only 19 samples were receptive. Of the patients with receptive samples who also underwent FET, 39% conceived. In addition, after further FET cycles, 50% of the patients with receptive samples were able to conceive. Lastly, after adjustments to the timing of FET were made for patients found to be pre-receptive or post-receptive, there was a pregnancy rate of 33.3%, which is comparable to the 35.2% pregnancy rate of the control group. Several studies have also indicated a clinical pregnancy rate of about 34% for non-ERA FET, thus showing this to be similar to the findings in the literature [64,65]. Since the pregnancy rates were comparable in both the control and ERA groups, the study concluded that having patients undergo ERA testing before FET shows no improvement in pregnancy rates in patients with zero or some implantation failures. While the literature does support this conclusion, the discrepancy in the size of the ERA and control groups should be taken into consideration.

Further retrospective studies that support this conclusion include one conducted from 2012 to 2018 by Neves et al. [62]. In this study, the effect of prior ERA testing on the implantation rate (IR) and pregnancy rate (PR) of patients with a history of failed embryo transfers was assessed. The data showed that there was no significant difference between the IR (55.6% vs. 65%) and PR (58.3% vs. 70.6%, *p* = 0.238) in the patient group that had undergone prior ERA testing versus the control group that had not. Additionally, a study conducted by Bergin et al. from 2014 to 2019 assessed the effect of performing an ERA on live birth rates [52]. The live birth rates for the ERA group and the non-ERA group were not statistically significant, being 49.62% and 54.96%, respectively, with an odds ratio of 0.8074 and a 95% confidence interval (0.5424–1.2018). Furthermore, the secondary outcomes included the positive pregnancy test rate (69.17% vs. 74.50%, *p* = 0.237), the clinical pregnancy rate (61.65% vs. 61.76, *p* = 0.984), and the miscarriage rate (8.27% vs. 6.52%, *p* = 0.499), which also were not statistically significant. Thus, the results of these studies reaffirm the conclusion that an ERA should not be routinely used prior to FET in unselected patient populations.

A prospective cohort study was reviewed consisting of patients that underwent their first FET at a private fertility center from January 2018 to April 2019 [53]. The purpose of this study was to assess if the standard use of an ERA in the first single-euploid FET cycles with an unselected population increases live birth rates. Prior to their first FET, patients were offered an ERA, but the decision of whether to undergo an ERA was up to the discretion of both the patient and the physician. Of the 228 frozen euploid embryo transfers, 81 were standard timing cycles, while 147 were ERA cycles. Out of the 147 patients undergoing ERA, 60 (40.8%) had receptive endometrial samples while 87 (59.2%) had nonreceptive samples. Of the nonreceptive endometrial samples,93.1%were preceptive and 6.9% were post-receptive. Notably, patients who had undergone prior ERA testing were older and thus had fewer euploid embryos present to transfer. Otherwise, the variables were controlled for BMI, endometrial lining thickness, and the used route for progesterone administration. The study concluded that there was no significant difference in live birth rate between patients who underwent ERA testing prior to FET (56.5%) and patients who received FET without ERA testing (56.6%). According to this data, ERA testing may not be an effective tool in an unselected patient population of individuals receiving their first single-euploid FET. Despite this conclusion, the literature on prospective studies is limited and thus further studies should be conducted.

Lastly, a randomized controlled trial was reviewed, occurring between November 2013 and April 2017, which compared the clinical outcomes of ERA-guided personalized embryo transfer (pET) to those of non-ERA FET or fresh blastocyst transfer [58]. Specifically, the pET group underwent hormone replacement therapy in the time period indicated by the ERA. The FET group, which was not ERA-guided, had exogenous progesterone administered for 5 days once serum progesterone levels were approximately 0. In the fresh embryo transfer group, the implantation occurred 5 to 6 days after the oocyte was retrieved based on blastocyst timing.

The clinical outcomes of this study show that the pregnancy rate for the first embryo transfer was 72.5% for the pET group compared to 54.3% (*p* = 0.01) in the group who had undergone FET and 58.5% (*p* = 0.05) in the group who had undergone fresh embryo transfer. After a follow-up period of 1 year, however, the cumulative pregnancy rate was higher in the pET group, at 93.6% compared to 79.7% (*p* = 0.0005) in the FET group and 80.7% (*p* = 0.0013) in the fresh embryo transfer group. The live birth rate for the first embryo transfer was 56.2% in the pET group compared to 42.4% (*p* = 0.09) in the FET group, and 45.7% (*p* = 0.17) in the fresh embryo transfer group. Alternatively, the cumulative live birth rate at the 1-year follow up was 71.2% in the pET group compared to 55.4% (*p* = 0.04) in the non-ERA FET group and 48.9% (*p* = 0.03) in the fresh embryo transfer group. Moreover, the implantation rate at the first embryo transfer was 57.3% in the pET group compared to 43.2% (*p* = 0.03) in non-ERA FET group, and 38.6% (*p* = 0.004) in the fresh embryo transfer group. Consequently, these results indicate a statistically significant increase in pregnancy, live birth rate, and implantation rates in the pET group. Even with the significant results in this study, the amount of literature on unselected populations utilizing ERA-guided embryo transfer is minimal, illustrating the need for future studies.

## 6. Clinical Outcomes Using ERA in Specific Populations

Given that the WOI has been demonstrated to shift in ¼ of women with RIF, it is unsurprising that much of the clinical data on the efficacy of the ERA has been evaluated in specific populations, including RIF patients, endometriosis patients, and adenomyosis patients [23]. In this section, we looked at studies that signify specific populations and if they demonstrate an advantage or disadvantage of using the ERA as a tool in their specific subset (Table 2).

In the case of both adenomyosis and endometriosis, many of the issues surrounding these conditions involve a pro-inflammatory state in the endometrium and an imbalance of estrogen and progesterone due to progesterone resistance [66,67]. This imbalance has been shown to decrease decidualization, which is an important factor in successful implantation. Decidualization provides nutrients to the embryo, protects against the maternal immune response, and regulates trophoblastic invasion. Instead, there is an evident estrogen predominance resulting in inflammation, increased cell proliferation, and overexpression of p450 aromatase, which has been associated with poor IVF outcomes [68].

RIF, specifically, remains an unaddressed clinical problem in many otherwise healthy women [69]. It is defined as the failure of three in vitro fertilization (IVF) cycles in which one or two morphologically high-grade embryos are transferred, following which special protocols must be enforced [70]. RIF falls into several larger clinical categories, one being issues with endometrial receptivity [71]. Prior studies have shown that there is a strong link between patients with RIF and alteration in endometrial receptivity [72]. One study demonstrated that in women with RIF, 303 genes defined the transcriptomic expression profile and were consistent with decreased cellular proliferation [73]. A further genomic study on women with RIF showed that there was an altered expression of 63 transcripts in the P +7 endometrium and dysregulation in 313 genes from endometrial samples on day 21 of the cycle in those with RIF in contrast to the samples of fertile women [74].

There is an increased percentage of WOI displacement in RIF patients, therefore leading to the incorporation of the ERA in personalized frozen embryo transfer (pET) as a therapeutic strategy. Multiple studies showed that when pET was employed in specifically RIF patients with a nonreceptive endometrium, the implantation rate and pregnancy rate increased to the level of that of receptive RIF patients [75,76,77,78,79]. A retrospective cohort study, performed in 2015 at a fertility center in India, demonstrates this as well [75]. In this study, Mahajan et al. investigated the ERA of RIF patients compared to that of non-RIF patients. They found that amongst the RIF patients, 27.5% (22/80) had an endometrial factor contributing to infertility. This was found to be statistically significant and greater than the number of patients in the non-RIF group having this factor, with only 15% (14/93) having an endometrial factor contributing to their infertility [75]. They performed an ERA and pET on both RIF and non-RIF subjects. Following the pET, the ongoing pregnancy rate for the non-RIF group was 56% (38/69) compared to 42.4% (28/66) for the RIF group. While the non-RIF group had a slightly higher pregnancy rate, the difference was statistically insignificant. The implantation rates for the RIF group and the non-RIF group were comparable as well, at 33% (42/129) and 39% (53/135), respectively. This is higher than the previously reported implantation rate for RIF patients of 10% in a similar study [80]. The study concluded that the ERA is a tool that is beneficial in RIF patients, increasing implantation and pregnancy rates to levels that are comparable to those of a non-RIF population.

Another retrospective cohort study investigated the efficacy of ERA testing in improving the ongoing/delivered pregnancy rates of patients with RIF (*n* = 24) [81]. This study found that 81% of their RIF patients had ERA results indicating that a modification of the transfer time interval was necessary to optimize their receptivity. Implementing the changes indicated by the ERA report, they had a 76.5% clinical pregnancy rate per transfer, a 50% implantation rate, and a 58.5% ongoing/delivery pregnancy rate per transfer. This study concluded that ERA testing was highly efficacious at achieving ongoing pregnancies amongst severe RIF patients [81]. In a further retrospective observational study, 248 patients with unexplained RIF underwent ERAs. It was found that the average number of failed IVF cycles was 3.67 ± 1.67 among receptive ERA patients and 4.09 ± 1.68 among nonreceptive ERA patients. Pregnancy rate, clinical PR, implantation rate, abortion rate, ongoing pregnancy rate, and cumulative PR were comparable between patients with a receptive ERA who had undergone a routine embryo transfer (ET) and those with a nonreceptive ERA who underwent a pET [38]. This study demonstrates the utility of the ERA in patients with RIF. A retrospective two-center study in Japan found similar results [37]. This study evaluated 50 patients with a history of RIF that underwent ERAs. Of these patients, 12 (24%) were nonreceptive and 8 (66.7%) were pre-receptive. The pregnancy rates were 58.8% per patient and 35.3% per first pET in the receptive patients, and 50% per patient and 50% per first pET in the nonreceptive patients. This study concluded that for patients with unexplained RIF, there is significance in searching for the pWOI and implementing these results in their FET protocol, given the pregnancy rates for nonreceptive patients. These aforementioned studies contribute to a formative body of work, which demonstrates the utility of the ERA in RIF patients.

An additional retrospective study evaluating 222 patients who underwent ERAs with prior failed FETs was performed. In total, 59% of the women had one or more failed FETs. They found that 45% of patients with at least one prior failed FET were nonreceptive. In this study, there was no difference between the pregnancy outcomes of those that had an ERA performed for them. This may provide insight that one failed embryo transfer may not be sufficient evidence for the use of the ERA [81,82]. Another retrospective multicenter cohort study was conducted in patients with RIF; patients were classified as having moderate or severe RIF based on the failed implantation of 3 or 5 embryos, respectively [83]. Of the 2110 patients in the moderate RIF group, those who underwent ERAs and transferred tested euploid embryos had the highest pregnancy rate compared to their counterparts. This study concluded that the ERA did not appear to significantly improve outcomes for either severe or recurrent RIF groups. However, there was a vast difference between those that had an ERA and those that did not for each group. For the moderate RIF group, 126 patients had an ERA compared to 1984 patients that did not have an ERA performed. For the severe RIF group, 27 patients had an ERA performed compared to 461 who did not have an ERA. This study demonstrates how necessary randomized control trials are, in addition to the need for more prospective studies to best determine the efficacy of the ERA.

A randomized control trial is currently being conducted to investigate the clinical efficacy of the ERA in Chinese patients with RIF; however, it is still ongoing. Alternatively, one prospective interventional multicenter clinical trial demonstrated the clinical value of the ERA in patients with RIF [23]. In total, 85 RIF patients were enrolled with 25 comparison patients. In this study, 74.1% of RIF patients were found to be receptive compared to the 88% of control subjects who were found to be receptive. Clinical follow up was only possible in 29 RIF patients, resulting in a 51.7% pregnancy rate and a 33.9% implantation rate. Of the RIF patients, 22 (25.9%) were found to be nonreceptive, and in 15 of these patients a second ERA was performed, confirming the WOI. In 8 of these patients, a pET was performed with adjustments indicated by the ERA, and there was a 50% pregnancy rate with a 38.5% implantation rate. These results are promising as the pregnancy and implantation rates of nonreceptive patients with an ERA were comparable to those of receptive patients. However, a major limitation of this study is the small sample size, with many patients having been lost due to the follow up. Therefore, it is necessary to perform additional prospective studies with a larger patient population to validate these preliminary results and build upon the very limited number of studies that currently exist on this topic.

In addition to RIF patients, there are other patient populations who may benefit from the ERA. Adenomyosis may have a negative impact on endometrial receptivity, reducing the probability of conception by assisted reproductive technology [84]. Through molecular analyses, this impairment in endometrial receptivity has been demonstrated in part to be due to the hyper-proliferation of the endometrial epithelium, impaired decidualization, progesterone resistance, altered expression of adhesion molecules, and a hyperinflammatory microenvironment [84]. One retrospective study showed the WOI to be displaced significantly in patients with adenomyosis (47.2%) compared with controls, making the risk ratio of a displaced WOI in adenomyosis as compared to controls to be 2:1 [60]. In this same study, the RIF rate was higher in adenomyosis patients compared to controls (66.6% and 34.9%, respectively). Following the use of the ERA and the implementation of the results in a pET, the pregnancy rate was 62.5% compared to that of 63.1% in the non-displaced WOI group with adenomyosis and that of 63.9% in patients without adenomyosis. This demonstrates endometrial receptivity and a displaced WOI as causes of implantation failure in patients with adenomyosis and RIF. This is seen as an improvement in the clinical pregnancy rate when compared to the rates reported in the literature. Similar studies have found the clinical pregnancy rate for women with adenomyosis achieved after IVF/ICSI to be 40.5%, which is significantly lower than the pregnancy rate found with an ERA [85,86]. An ERA is therefore helpful in identifying the WOI, and when incorporated in pET in patients with RIF may improve implantation rates and pregnancy outcomes [38]. A limitation of this comparison is that to the best of our knowledge, there are no other studies investigating adenomyosis and the ERA that currently exist.

While there are improved outcomes in patients with RIF and adenomyosis shown in the literature, this does not appear to be the case with endometriosis patients. Many believed that endometrial receptivity, specifically displacement of the WOI, would be an issue for patients with endometriosis given the chronic inflammatory processes leading to both infertility and heavy bleeding in this condition [66]. However, when endometrial biopsies from those with endometriosis were compared to those from controls, none of the 238 genes present in the ERA were significantly under- or over-expressed [87]. This suggests that the endometrial receptivity gene signature may not vary the window of implantation in endometriosis; however, there may be other endometrial factors at play in disease progression and the presentation of infertility [87]. This is unexpected, given that the data from the aforementioned study identified improved outcomes in patients experiencing adenomyosis with RIF, and given the similarities between adenomyosis and endometriosis. Regardless, to the best of our knowledge, there are no current studies investigating the ERA in patients with endometriosis, and thus we cannot whether or not ERA may be an asset in this patient population. Furthermore, for adenomyosis-specific studies, it is difficult to state definitively whether the improved outcomes are a result of these patients coincidentally having higher rates of RIF and thus some alternative underlying cause or a result of adenomyosis pathogenesis. Therefore, it is necessary that we perform further studies investigating ERA use in patients with adenomyosis. Nonetheless, the fact that there are statistically significant improved outcomes of incorporating an ERA in FET protocols for RIF patients is promising.

**Table 2:** Embryo transfer outcomes in patients with RIF and adenomyosis. The clinical outcomes of the studies for specific populations in this review are listed in the order they were discussed. The table compares the implantation and pregnancy rates in repeated implantation failure and adenomyosis populations. The study including adenomyosis populations also examined the efficacy of the ERA on pregnancy rates. ERA = endometrial receptivity array. Where N/A is indicated, notes that the data for the specified variable was not offered in the given study.

Study Type	Implantation Rate (RIF)	Implantation Rate (Non-RIF)	Pregnancy Rate (RIF)	Pregnancy Rate (Non-RIF)	Pregnancy Rate (Adenomyosis)	Pregnancy Rate (No Adenomyosis)
Retrospective cohort study [78]	33%	39%	42.4% ongoing	56%	N/A	N/A
Retrospective cohort study [84]	50%	N/A	58.5% ongoing, 76.5% clinical	N/A	N/A	N/A
Retrospective cohort study [38]	N/A	N/A	58.8% receptive patients, 50% nonreceptive patients	N/A	N/A	N/A
Prospective case–control study [23]	33.9%	N/A	51.7%	N/A	N/A	N/A
Retrospective cohort study [88]	N/A	N/A	N/A	N/A	62.5% with ERA, 63.1% without ERA	63.9%

## 7. Summary and Discussion

The ERA is a tool used to assess endometrial receptivity. The ERA involves an endometrial biopsy and can be performed concurrently with an additional endometrial biopsy for pathological review. The ERA uses molecular arrays to assess proliferation and differentiation in the endometrium and “date” the endometrium [6]. The ERA was first developed in 2011 by Diaz-Gimeno et al. who identified 238 genes that were differentially expressed at the receptive phase and published their transcriptomic analysis [17]. In the development of this tool, they utilized endometrium samples from fertile women to train the predictor for endometrial dating, which showed a specificity of 0.8857 and a sensitivity of 0.99758 for endometrial dating. Thus, the ERA is clearly an efficacious tool that can be used to determine endometrial receptivity status.

While WOI length is constant amongst most women, it is displaced in 20% of the population and in ¼ of women with RIF [23]. Therefore, the ERA can aid in identifying a personalized WOI which can then be applied to a patient’s FET plan, to increase the chances of a successful transfer [17]. The ERA can be used in the setting of both HRT-FET and nFET. When using an ERA, a patient will ultimately need two cycles, the first being an ERA cycle and the second being a nFET or HRT-FET cycle [23,52,53]. In the ERA cycle, the patient takes the same medications they would in a HRT-FET cycle or a modified nFET cycle; however, rather than embryo transfer occurring, an endometrial biopsy can be performed and sent for an ERA. In the case of HRT-FET, the endometrial biopsy is performed on day P +5 and in nFET, the biopsy is taken either at LH +7 or hCG +7. Following the endometrial biopsy, the ERA report can be returned with one of three results: receptive, pre-receptive, or post-receptive. If the result is receptive, then the ERA results are used to determine the date of embryo transfer and/or the initiation of the administration of progesterone or bhCG depending on whether a patient is undergoing HRT-FET or nFET, respectively. If the result is nonreceptive, then it can be either pre-receptive or post-receptive. In this case, Igenomix recommends conducting a repeat ERA, shifting the time of the biopsy accordingly, so that the patient will be receptive. However, it is up to the discretion of the provider and patient whether to repeat the biopsy or rather calculate an approximated WOI based on the results of the first ERA.

In a review of the clinical outcomes of the use of the ERA, it was evident that in a patient population of infertile women that did not meet the criteria of RIF, there was no statistically significant benefit of performing an ERA. Some studies did demonstrate a slight increase in live birth rates and decreased miscarriage rates in patients with first-time FETs, but the data was again not significant.

However, in the case of RIF patients and patients with adenomyosis, there seems to be a benefit in using ERA. Multiple studies have shown that when pET was employed in specifically RIF patients with a nonreceptive endometrium, the implantation rate and pregnancy rate increased to the level of receptive RIF patients. Further studies have shown the WOI to be displaced significantly in adenomyosis (47.2%) compared with controls [60]. An ERA is therefore helpful in identifying the WOI, and when incorporated in pET in patients with RIF may improve implantation rates and pregnancy outcomes [38]. While there are improved outcomes in patients with RIF and adenomyosis shown in the literature, this does not appear to be the case with endometriosis patients. Reviewing the improved implantation rates and pregnancy outcomes in these patients, it is important to consider that these patients may need to undergo fewer fertility treatments, if their goals are accomplished sooner.

In potentially reducing the number of fertility treatments a patient may need to undergo the psycho-emotional exhaustion involved in undergoing additional treatments may be mitigated [88]. In addition to relieving some of the psychological burden of undergoing additional fertility treatments, some of the financial burden may be relieved as well. Following failed embryo transfer, the patient often has a couple of options. The first option would be to transfer additional embryos if the patient has other embryos stored. The second option would be to undergo additional egg retrievals as a component of their IVF cycle to create and store embryos, replenishing their depleted store. Both of these options incur significant costs to the patient [89,90].

The ERA has been shown to cost around 850 to 1000 US at various institutions, and it may or may not be covered by insurance [91,92]. While it comes at a substantial cost, these costs are better compared to the additional 3500 to 6350 USD for a repeat FET if the initial cycle fails, or 15,000 to 30,000 USD for an entire IVF cycle altogether if further embryos need to be made, depending on the center and medication protocol of the patient [93,94,95]. Therefore, patients who are good candidates for the ERA may be able to save tens of thousands of dollars by requiring fewer cycles. Thus, using the ERA for improving implantation rates and pregnancy outcomes in certain populations may offer relief, decreasing the burden for patients across the board. This is not to say that the ERA will so improve these rates in all patient populations; however, it is a tool that if applied in appropriate cases can be incredibly powerful.

With the development of the ERA, there is a lot of dissent in the field challenging its efficacy. Some doubt the need for the ERA at all. Several clinicians argue that the implantation window is at least three days in duration; therefore, precision in the timing of embryo transfer is entirely unnecessary [8,96]. While that fact is true, it has been demonstrated previously that this window can be entirely shifted out of the three-day frame altogether, thus determining that this timeframe is far from unnecessary. Others doubt the methodology that the ERA employs. Due to the lack of histological analysis in the ERA, certain pathologies that play a role may be missed [97,98]. While these doubts are justified, an additional endometrial biopsy can be taken concurrently at the time of the ERA and sent for pathological review, which is often covered by insurance. Alternatively, if the ERA were to expand its analysis to cover histological analysis, it would inevitably be more costly and oftentimes not covered by insurance, thus possibly decreasing the accessibility of this tool. Other people challenge the fact that while the ERA “dates” the endometrium, dating alone is not sufficient to discriminate between fertile and infertile women [99]. However, the function of the ERA implies that it is to be used primarily in infertile women, and thus its inability to be used in this population is not a major drawback. Lastly, it is indisputable that the randomized control trials show that when the ERA is applied in unspecified populations, there is no significant difference in implantation rates and pregnancy rates among these women. Based on these findings, the ERA may not be advantageous to the general population of people seeking fertility treatment.

However, as important as it is to identify populations in which the ERA may not be effective, it is equally as important to aim our focus at identifying those who the ERA may help. While it is true that it may not be used in all circumstances, there is clear evidence that in patients with RIF, there are statistical differences noted in improved pregnancy outcomes. Nevertheless, the data are limited, as there has not yet been a randomized control trial investigating the use of the ERA in women with RIF. Further studies are needed to better understand the populations in which the ERA may be beneficial, possibly supporting the current clinical data available. Through additional randomized controlled trials demonstrating the use of the ERA, we hope that guidelines can be established so that providers can have guidance on when the ERA is most appropriate.

## 8. Conclusions

It is evident that the ERA is a novel technology that assesses and provides a vast amount of information about a patient’s endometrial receptivity. From a provider’s standpoint, the analysis requires only an endometrial biopsy to be performed. While the ERA may not improve outcomes in the case of every patient seeking fertility treatment, there is a clear benefit for specific populations, as demonstrated in studies investigating its use in RIF and adenomyosis patients. In these settings, an ERA can offer many benefits. In the review of ERA, the settings in which it can be used, and its efficacy in several different populations, it is clear that the focus of the conversation should be less on whether or not the ERA is an effective tool but instead focus on when it can be used effectively.

## Figures and Tables

**Table 1:** Embryo transfer outcomes in infertile patients. The clinical outcomes of the studies for infertile women in this review are listed in the order they were discussed. The table compares the clinical outcomes for embryo transfers occurring with or without ERA use and PGT-A. ERA = endometrial receptivity array, PGT-A = pre-implantation genetic testing for aneuploidy.

Retrospective Cohort Study: No Prior Genetic Screening [57]			
Variable	Non-ERA Group	ERA Group	
Live birth rate	39.7%	50%	
Miscarriage rate	10.7%	5.6%	
**Retrospective Cohort Studies: With Prior Genetic Screening**			
Study 1 [58]			
**Variable**	**Non-ERA Group**	**ERA Group (Receptive Only)**	**ERA Group (With Adjustment)**
Pregnancy rate	35.2%	39%	33.3%
Study 2 [59]			
**Variable**	**Non-ERA Group**	**ERA Group**	
Implantation rate	65%	55.6%	
Pregnancy rate	70%	58.3%	
Study 3 [54]:			
**Variable**	**Non-ERA Group**	**ERA Group**	
Live birth rate	54.96%	49.62%	
Positive pregnancy test rate	74.50%	69.17%	
Clinical pregnancy rate	61.76%	61.65%	
Miscarriage rate	6.52%	8.27%	
**Prospective Cohort Study: With Prior Genetic Screening [55]**			
**Variable**	**Non-ERA Group**	**ERA Group**	
Live birth rate	56.6%	56.5%	
**Randomized Controlled Trial: With Prior Genetic Screening [60]**			
**Variable**	**Non-ERA Group (Fresh Embryo Transfer)**	**Non-ERA Group (FET)**	**ERA Group (pET)**
Pregnancy rate (first embryo transfer)	58.5%	54.3%	72.5%
Pregnancy rate (cumulative)	80.7%	79.7%	93.6%
Live birth rate (first embryo transfer)	45.7%	42.4%	56.2%
Live birth rate (cumulative)	48.9%	55.4%	71.2%
Implantation rate	38.6%	43.2%	57.3%

## References

[1] Scott R.T., Ferry K., Su J., Tao X., Scott K., Treff N.R. (2012). Comprehensive chromosome screening is highly predictive of the reproductive potential of human embryos: A prospective, blinded, nonselection study. Fertil. Steril..

[2] Heger A., Sator M., Pietrowski D. (2012). Endometrial Receptivity and its Predictive Value for IVF/ICSI-Outcome. Geburtshilfe Und Frauenheilkd..

[3] Penzias A., Bendikson K., Butts S., Coutifaris C., Falcone T., Fossum G., Gitlin S., Gracia C., Hansen K., La Barbera A. (2018). The use of preimplantation genetic testing for aneuploidy (PGT-A): A committee opinion. Fertil. Steril..

[4] Simopoulou M., Sfakianoudis K., Maziotis E., Tsioulou P., Grigoriadis S., Rapani A., Giannelou P., Asimakopoulou M., Kokkali G., Pantou A. (2021). PGT-A: Who and when? A systematic review and network meta-analysis of RCTs. J. Assist. Reprod. Genet..

[5] Twisk M., Mastenbroek S., van Wely M., Heineman M.J., Van Der Veen F., Repping S. (2006). Preimplantation genetic screening for abnormal number of chromosomes (aneuploidies) in in vitro fertilisation or intracytoplasmic sperm injection. Cochrane Database Syst. Rev..

[6] Kliman H.J., Frankfurter D. (2019). Clinical approach to recurrent implantation failure: Evidence-based evaluation of the endometrium. Fertil. Steril..

[7] Achache H., Revel A. (2006). Endometrial receptivity markers, the journey to successful embryo implantation. Hum. Reprod. Update.

[8] Lessey B.A., Young S.L. (2019). What exactly is endometrial receptivity?. Fertil. Steril..

[9] Moustafa S., Young S. (2020). Diagnostic and therapeutic options in recurrent implantation failure. F1000Research.

[10] Dorostghoal M., Ghaffari H.-O., Moramezi F., Keikhah N. (2018). Overexpression of Endometrial Estrogen Receptor-Alpha in The Window of Implantation in Women with Unexplained Infertility. Int. J. Fertil. Steril..

[11] Noyes R.W., Hertig A.T., Rock J. (1975). Dating the endometrial biopsy. Am. J. Obstet. Gynecol..

[12] Kliman H.J., McSweet J.C., Grunert G.M., Cardone V.R., Cadesky K., Keefe D.L. (2002). The endometrial function test (EFT) directs care and predicts ART outcome. Fertil. Steril..

[13] Kliman H.J., Honig S., Walls D., Luna M., McSweet J.C., Copperman A.B. (2006). Optimization of endometrial preparation results in a normal endometrial function test^®^ (EFT^®^) and good reproductive outcome in donor ovum recipients. J. Assist. Reprod. Genet..

[14] Soriano D., Adler I., Bouaziz J., Zolti M., Eisenberg V.H., Goldenberg M., Seidman D.S., Elizur S.E. (2016). Fertility outcome of laparoscopic treatment in patients with severe endometriosis and repeated in vitro fertilization failures. Fertil. Steril..

[15] Littman E., Giudice L., Lathi R., Berker B., Milki A., Nezhat C. (2005). Role of laparoscopic treatment of endometriosis in patients with failed in vitro fertilization cycles. Fertil. Steril..

[16] Macer M.L., Taylor H.S. (2012). Endometriosis and Infertility: A Review of the Pathogenesis and Treatment of Endometriosis-associated Infertility. Obstet. Gynecol. Clin. N. Am..

[17] Díaz-Gimeno P., Horcajadas J.A., Martinez-Conejero J.A., Esteban F.J., Alama P., Pellicer A., Simon C. (2011). A genomic diagnostic tool for human endometrial receptivity based on the transcriptomic signature. Fertil. Steril..

[18] Venter J.C., Adams M.D., Myers E.W., Li P.W., Mural R.J., Sutton G.G., Smith H.O., Yandell M., Evans C.A., Holt R.A. (2001). The Sequence of the Human Genome. Science.

[19] Gómez E., Ruíz-Alonso M., Miravet J., Simón C. (2015). Human Endometrial Transcriptomics: Implications for Embryonic Implantation. Cold Spring Harb. Perspect. Med..

[20] Altmäe S., Esteban F.J., Stavreus-Evers A., Simón C., Giudice L., Lessey B.A., Horcajadas J.A., Macklon N.S., D’Hooghe T., Campoy C. (2014). Guidelines for the design, analysis and interpretation of ‘omics’ data: Focus on human endometrium. Hum. Reprod. Update.

[21] Ruiz-Alonso M., Blesa D., Simón C. (2012). The genomics of the human endometrium. Biochim. Biophys. Acta BBA-Mol. Basis Dis..

[22] Díaz-Gimeno P., Ruiz-Alonso M., Blesa D., Bosch N., Martínez-Conejero J.A., Alamá P., Garrido N., Pellicer A., Simón C. (2013). The accuracy and reproducibility of the endometrial receptivity array is superior to histology as a diagnostic method for endometrial receptivity. Fertil. Steril..

[23] Garrido-Gómez T., Ruiz-Alonso M., Blesa D., Diaz-Gimeno P., Vilella F., Simón C. (2013). The endometrial receptivity array for diagnosis and personalized embryo transfer as a treatment for patients with repeated implantation failure. Fertil. Steril..

[24] Wong K.M., Mastenbroek S., Repping S. (2014). Cryopreservation of human embryos and its contribution to in vitro fertilization success rates. Fertil. Steril..

[25] Zhang J., Du M., Li Z., Wang L., Hu J., Zhao B., Feng Y., Chen X., Sun L. (2018). Fresh versus frozen embryo transfer for full-term singleton birth: A retrospective cohort study. J. Ovarian Res..

[26] Shapiro B.S., Daneshmand S.T., Garner F.C., Aguirre M., Hudson C., Thomas S. (2011). Evidence of impaired endometrial receptivity after ovarian stimulation for in vitro fertilization: A prospective randomized trial comparing fresh and frozen–thawed embryo transfer in normal responders. Fertil. Steril..

[27] Bhattacharya S. (2016). Maternal and perinatal outcomes after fresh versus frozen embryo transfer—What is the risk-benefit ratio?. Fertil. Steril..

[28] Zhao J., Xu B., Zhang Q., Li Y.P. (2016). Which one has a better obstetric and perinatal outcome in singleton pregnancy, IVF/ICSI or FET?: A systematic review and meta-analysis. Reprod. Biol. Endocrinol..

[29] Evans J., Hannan N.J., Edgell T.A., Vollenhoven B.J., Lutjen P.J., Osianlis T., Salamonsen L.A., Rombauts L.J.F. (2014). Fresh versus frozen embryo transfer: Backing clinical decisions with scientific and clinical evidence. Hum. Reprod. Update.

[30] Blanco-Breindel M.F., Singh M., Kahn J. (2023). Endometrial Receptivity. StatPearls [Internet].

[31] Groenewoud E., Cohlen B., Al-Oraiby A., Brinkhuis E., Broekmans F., de Bruin J., Dool G.V.D., Fleisher K., Friederich J., Goddijn M. (2016). A randomized controlled, non-inferiority trial of modified natural versus artificial cycle for cryo-thawed embryo transfer. Hum. Reprod..

[32] Agha-Hosseini M., Hashemi L., Aleyasin A., Ghasemi M., Sarvi F., Nashtaei M.S., Khodarahmian M. (2018). Natural cycle versus artificial cycle in frozen-thawed embryo transfer: A randomized prospective trial. J. Turk. Soc. Obstet. Gynecol..

[33] Mumusoglu S., Polat M., Ozbek I.Y., Bozdag G., Papanikolaou E.G., Esteves S.C., Humaidan P., Yarali H. (2021). Preparation of the Endometrium for Frozen Embryo Transfer: A Systematic Review. Front. Endocrinol..

[34] Eleftheriadou A., Francis A., Wilcox M., Jayaprakasan K. (2022). Frozen Blastocyst Embryo Transfer: Comparison of Protocols and Factors Influencing Outcome. J. Clin. Med..

[35] Greenbaum S., Athavale A., Klement A.H., Bentov Y. (2022). Luteal phase support in fresh and frozen embryo transfers. Front. Reprod. Health.

[36] Jia Y., Sha Y., Qiu Z., Guo Y., Tan A., Huang Y., Zhong Y., Dong Y., Ye H. (2022). Comparison of the Effectiveness of Endometrial Receptivity Analysis (ERA) to Guide Personalized Embryo Transfer with Conventional Frozen Embryo Transfer in 281 Chinese Women with Recurrent Implantation Failure. Med. Sci. Monit. Int. Med. J. Exp. Clin. Res..

[37] Hashimoto T., Koizumi M., Doshida M., Toya M., Sagara E., Oka N., Nakajo Y., Aono N., Igarashi H., Kyono K. (2017). Efficacy of the endometrial receptivity array for repeated implantation failure in Japan: A retrospective, two-centers study. Reprod. Med. Biol..

[38] Patel J.A., Patel A.J., Banker J.M., Shah S.I., Banker M.R. (2019). Personalized embryo transfer helps in improving In vitro fertilization/ICSI outcomes in patients with recurrent implantation failure. J. Hum. Reprod. Sci..

[39] ERA-EMMA-ALICE-Manual-EndomeTRIO-Manual-USA-2021.pdf [Internet]. https://www.igenomix.ca/wp-content/uploads/sites/14/2021/10/ERA-EMMA-ALICE-Manual-EndomeTRIO-Manual-USA-2021.pdf.

[40] Madero S., Rodriguez A., Vassena R., Vernaeve V. (2016). Endometrial preparation: Effect of estrogen dose and administration route on reproductive outcomes in oocyte donation cycles with fresh embryo transfer. Hum. Reprod..

[41] Glujovsky D., Pesce R., Fiszbajn G., Sueldo C., Hart R.J., Ciapponi A. (2020). Endometrial preparation for women undergoing embryo transfer with frozen embryos or embryos derived from donor oocytes. Cochrane Database Syst. Rev..

[42] Devroey P., Pados G. (1998). Preparation of endometrium for egg donation. Hum. Reprod. Update.

[43] Navot D., Laufer N., Kopolovic J., Rabinowitz R., Birkenfeld A., Lewin A., Granat M., Margalioth E.J., Schenker J.G. (1986). Artificially Induced Endometrial Cycles and Establishment of Pregnancies in the Absence of Ovaries. N. Engl. J. Med..

[44] Borini A., Prato L.D., Bianchi L., Violini F., Cattoli M., Flamigni C. (2001). CLINICAL ASSISTED REPRODUCTION: Effect of Duration of Estradiol Replacement on the Outcome of Oocyte Donation. J. Assist. Reprod. Genet..

[45] Bourdon M., Santulli P., Maignien C., Gayet V., Pocate-Cheriet K., Marcellin L., Chapron C. (2018). The deferred embryo transfer strategy improves cumulative pregnancy rates in endometriosis-related infertility: A retrospective matched cohort study. PLoS ONE.

[46] Sekhon L., Feuerstein J., Pan S., Overbey J., Lee J.A., Briton-Jones C., Flisser E., Stein D.E., Mukherjee T., Grunfeld L. (2019). Endometrial preparation before the transfer of single, vitrified-warmed, euploid blastocysts: Does the duration of estradiol treatment influence clinical outcome?. Fertil. Steril..

[47] Zarei A., Sohail P., Parsanezhad M.E., Alborzi S., Samsami A., Azizi M. (2016). Comparison of four protocols for luteal phase support in frozen-thawed Embryo transfer cycles: A randomized clinical trial. Arch. Gynecol. Obstet..

[48] Tabibzadeh S. (1998). Molecular control of the implantation window. Hum. Reprod. Update.

[49] Franasiak J.M., Ruiz-Alonso M., Scott R.T., Simón C. (2016). Both slowly developing embryos and a variable pace of luteal endometrial progression may conspire to prevent normal birth in spite of a capable embryo. Fertil. Steril..

[50] Weissman A. (2017). RESULTS—Frozen-Thawed Embryo Transfer—IVF-Worldwide [Internet]. https://ivf-worldwide.com/survey/frozen-thawed-embryo-transfer/results-frozen-thawed-embryo-transfer.html.

[51] Escribá M.-J., Bellver J., Bosch E., Sánchez M., Pellicer A., Remohí J. (2006). Delaying the initiation of progesterone supplementation until the day of fertilization does not compromise cycle outcome in patients receiving donated oocytes: A randomized study. Fertil. Steril..

[52] Bergin K., Eliner Y., Duvall D.W., Roger S., Elguero S., Penzias A.S., Sakkas D., Vaughan D.A. (2021). The use of propensity score matching to assess the benefit of the endometrial receptivity analysis in frozen embryo transfers. Fertil. Steril..

[53] Riestenberg C., Kroener L., Quinn M., Ching K., Ambartsumyan G. (2021). Routine endometrial receptivity array in first embryo transfer cycles does not improve live birth rate. Fertil. Steril..

[54] Personalized Embryo Transfer (PET) Calculator. Igenomix. (n.d.). https://clinics.myigenomix.com/pet/calculator.

[55] Steptoe P.C., Edwards R.G. (1976). Reimplantation of a Human Embryo with Subsequent Tubal Pregnancy. Lancet.

[56] Yang Z., Liu J., Collins G.S., Salem S.A., Liu X., Lyle S.S., Peck A.C., Sills E.S., Salem R.D. (2012). Selection of single blastocysts for fresh transfer via standard morphology assessment alone and with array CGH for good prognosis IVF patients: Results from a randomized pilot study. Mol. Cytogenet..

[57] Kort J.D., McCoy R.C., Demko Z., Lathi R.B. (2018). Are blastocyst aneuploidy rates different between fertile and infertile populations?. J. Assist. Reprod. Genet..

[58] A 5-Year Multicentre Randomized Controlled Trial Comparing Personalized, Frozen and Fresh Blastocyst Transfer in IVF-Reproductive BioMedicine Online [Internet]. https://www.rbmojournal.com/article/S1472-6483(20)30319-9/fulltext.

[59] Bassil R., Casper R., Samara N., Hsieh T.B., Barzilay E., Orvieto R., Haas J. (2018). Does the endometrial receptivity array really provide personalized embryo transfer?. J. Assist. Reprod. Genet..

[60] Mahajan N., Kaur S., Alonso M.R. (2018). Window of implantation is significantly displaced in patients with adenomyosis with previous implantation failure as determined by endometrial receptivity assay. J. Hum. Reprod. Sci..

[61] Hombalegowda R., Ziegler W. (2020). Evaluating the role of endometrial receptivity array (era) in patients with first Frozen Embryo Transfers (FET). Fertil. Steril..

[62] Neves A.R., Devesa M., Martínez F., Garcia-Martinez S., Rodriguez I., Polyzos N.P., Coroleu B. (2019). What is the clinical impact of the endometrial receptivity array in PGT-A and oocyte donation cycles?. J. Assist. Reprod. Genet..

[63] Brosens J.J., Salker M.S., Teklenburg G., Nautiyal J., Salter S., Lucas E.S., Steel J.H., Christian M., Chan Y.-W., Boomsma C.M. (2014). Uterine Selection of Human Embryos at Implantation. Sci. Rep..

[64] Maheshwari A., Bari V., Bell J.L., Bhattacharya S., Bhide P., Bowler U., Brison D., Child T., Chong H.Y., Cheong Y. (2022). Transfer of thawed frozen embryo versus fresh embryo to improve the healthy baby rate in women undergoing IVF: The E-Freeze RCT. Health Technol. Assess..

[65] Atkinson M., Crittenden J., Smith H., Sjoblom C. (2021). Retrospective cohort study on preparation regimens for frozen embryo transfer. Reprod. Fertil..

[66] Lessey B.A., Kim J.J. (2017). Endometrial Receptivity in Eutopic Endometrium of Women with Endometriosis It is affected, let me show you why. Fertil. Steril..

[67] Fox C., Morin S., Jeong J.W., Scott R.T., Lessey B.A. (2016). Local and systemic factors and implantation: What is the evidence?. Fertil. Steril..

[68] Brosens J., Verhoeven H., Campo R., Gianaroli L., Gordts S., Hazekamp J., Hägglund L., Mardesic T., Varila E., Zech J. (2004). High endometrial aromatase P450 mRNA expression is associated with poor IVF outcome. Hum. Reprod..

[69] Repeated Implantation Failure: Clinical Approach-Fertility and Sterility [Internet]. https://www.fertstert.org/article/S0015-0282(12)00324-X/fulltext.

[70] Stephenson M.D., Fluker M.R. (2000). Treatment of repeated unexplained in vitro fertilization failure with intravenous immunoglobulin: A randomized, placebo-controlled Canadian trial. Fertil. Steril..

[71] Meyer W.R., Castelbaum A.J., Somkuti S., Sagoskin A.W., Doyle M., Harris J.E., Lessey B.A. (1997). Hydrosalpinges adversely affect markers of endometrial receptivity. Hum. Reprod..

[72] Li T.C., Klentzeris L., Barratt C., Warren M.A., Cooke S., Cooke I.D. (1993). A study of endometrial morphology in women who failed to conceive in a donor insemination programme. BJOG Int. J. Obstet. Gynaecol..

[73] Koot Y.E.M., van Hooff S.R., Boomsma C.M., van Leenen D., Groot Koerkamp M.J.A., Goddijn M., Eijkemans M.J.C., Fauser B.C.J.M., Holstege F.C.P., Macklon N.S. (2016). An endometrial gene expression signature accurately predicts recurrent implantation failure after IVF. Sci. Rep..

[74] Tapia A., Gangi L.M., Zegers-Hochschild F., Balmaceda J., Pommer R., Trejo L., Pacheco I.M., Salvatierra A.M., Henríquez S., Quezada M. (2007). Differences in the endometrial transcript profile during the receptive period between women who were refractory to implantation and those who achieved pregnancy. Hum. Reprod..

[75] Mahajan N. (2015). Endometrial receptivity array: Clinical application. J. Hum. Reprod. Sci..

[76] Tan J., Kan A., Hitkari J., Taylor B., Tallon N., Warraich G., Yuzpe A., Nakhuda G. (2018). The role of the endometrial receptivity array (ERA) in patients who have failed euploid embryo transfers. J. Assist. Reprod. Genet..

[77] Ota T., Funabiki M., Tada Y., Karita M., Hayashi T., Maeda K., Matsubara T., Iwaki Y., Sugiyama N., Henmi T. (2019). The Reproductive Outcomes for the Infertile Patients with Recurrent Implantation Failures May Be Improved by Endometrial Receptivity Array Test. J. Med. Cases.

[78] Kaur S., Naidu P. (2019). Why results of endometrial receptivity assay testing should not be discounted in recurrent implantation failure?. Onco Fertil. J..

[79] Kasahara Y., Hashimoto T., Yokomizo R., Takeshige Y., Yoshinaga K., Toya M., Igarashi H., Kishi H., Kyono K. (2021). Evaluation of Pregnancy Outcomes of Vitrified-Warmed Blastocyst Transfer before and after Endometrial Receptivity Analysis in Identical Patients with Recurrent Implantation Failure. Fertil. Reprod..

[80] Ocal P., Cift T., Bulut B., Balcan E., Cepni I., Aydogan B., Irez T. (2012). Recurrent Implantation Failure Is More Frequently Seen in Female Patients with Poor Prognosis. Int. J. Fertil. Steril..

[81] Rose B.I., Cover L., Brown S. (2020). On the utility of the endometrial receptivity assay (era) to correct recurrent implantation failure (rif): Not as simple as it seems. Fertil. Steril..

[82] Eisman L.E., Pisarska M.D., Wertheimer S., Chan J.L., Akopians A.L., Surrey M.W., Danzer H.C., Ghadir S., Chang W.Y., Alexander C.J. (2021). Clinical utility of the endometrial receptivity analysis in women with prior failed transfers. J. Assist. Reprod. Genet..

[83] Cozzolino M., Diaz-Gimeno P., Pellicer A., Garrido N. (2020). Evaluation of the endometrial receptivity assay and the preimplantation genetic test for aneuploidy in overcoming recurrent implantation failure. J. Assist. Reprod. Genet..

[84] Hiraoka T., Hirota Y., Osuga Y. (2022). How does adenomyosis impact endometrial receptivity? An updated systematic review of clinical and molecular insights. F&S Rev..

[85] Vercellini P., Consonni D., Dridi D., Bracco B., Frattaruolo M.P., Somigliana E. (2014). Uterine adenomyosis and in vitro fertilization outcome: A systematic review and meta-analysis. Hum. Reprod..

[86] Squillace A.L., Simonian D.S., Allegro M.C., Júnior E.B., Bianchi P.H.D.M., Bibancos M. (2021). Adenomyosis and in vitro fertilization impacts—A literature review. JBRA Assist. Reprod..

[87] Garcia-Velasco J.A., Fassbender A., Ruiz-Alonso M., Blesa D., D‘hooghe T., Simon C. (2015). Is endometrial receptivity transcriptomics affected in women with endometriosis? A pilot study. Reprod. Biomed. Online.

[88] Cousineau T.M., Domar A.D. (2007). Psychological impact of infertility. Best Practice Res. Clin. Obstet. Gynaecol..

[89] Coughlan C. (2018). What to do when good-quality embryos repeatedly fail to implant. Best Practice Res. Clin. Obstet. Gynaecol..

[90] Santos-Ribeiro S., Siffain J., Polyzos N.P., van de Vijver A., van Landuyt L., Stoop D., Tournaye H., Blockeel C. (2016). To delay or not to delay a frozen embryo transfer after a failed fresh embryo transfer attempt?. Fertil. Steril..

[91] Brogaard K. (2018). What Is an Endometrial Receptivity Array (ERA)? [Internet]. Path Fertility. https://pathfertility.com/what-is-an-endometrial-receptivity-array-era/.

[92] (2021). What Is ERA? Do I Need an ERA Test for Successful IVF? [Internet]. RMA Network-Fertility Clinic. https://rmanetwork.com/blog/era-test-for-ivf-endometrial-receptivity-analysis/.

[93] ColoCRM. How Much Does IVF Cost?|IVF Costs [Internet]. CCRM Fertility. https://www.ccrmivf.com/ivf-cost/.

[94] How Much Does IVF Cost In 2023?—Forbes Health [Internet]. https://www.forbes.com/health/family/how-much-does-ivf-cost/.

[95] The Cost of IVF in 2022 [Internet]. https://fertilityspace.io/blog/the-cost-of-ivf-in-2022.

[96] Bergh P.A., Navot D. (1992). The impact of embryonic development and endometrial maturity on the timing of implantation. Fertil. Steril..

[97] Cohen A.M., Ye X.Y., Colgan T.J., Greenblatt E.M., Chan C. (2020). Comparing endometrial receptivity array to histologic dating of the endometrium in women with a history of implantation failure. Syst. Biol. Reprod. Med..

[98] Endometrial Receptivity Revisited: Endometrial Transcriptome Adjusted for Tissue Cellular Heterogeneity|Human Reproduction|Oxford Academic [Internet]. https://academic.oup.com/humrep/article/33/11/2074/5123535?login=false.

[99] Coutifaris C., Myers E.R., Guzick D.S., Diamond M.P., Carson S.A., Legro R.S., McGovern P.G., Schlaff W.D., Carr B.R., Steinkampf M.P. (2004). Histological dating of timed endometrial biopsy tissue is not related to fertility status. Fertil. Steril..

